# What about drinking is associated with shorter life in poorer people?

**DOI:** 10.1371/journal.pmed.1002477

**Published:** 2018-01-02

**Authors:** Jürgen Rehm, Charlotte Probst

**Affiliations:** 1 Institute for Mental Health Policy Research, CAMH, Toronto, Ontario, Canada; 2 Campbell Family Mental Health Research Institute, CAMH, Toronto, Ontario, Canada; 3 Institute of Medical Science (IMS), University of Toronto, Toronto, Ontario, Canada; 4 Department of Psychiatry, University of Toronto, Toronto, Ontario, Canada; 5 Dalla Lana School of Public Health, University of Toronto, Toronto, Ontario, Canada; 6 Institute for Clinical Psychology and Psychotherapy, TU Dresden, Dresden, Germany

## Abstract

In a Perspective, Jürgen Rehm and Charlotte Probst examine the links between socioeconomic status, alcohol use, and cardiovascular mortality and discuss implications for policy.

## Income inequalities and the role of risk factors

Income inequalities and associated health consequences—in particular, premature mortality—are a major public health problem in the 21st century [1]. Poorer people die earlier, and they die in part of different causes of death. While the differential distributions of risk factors by socioeconomic status (SES) contribute to the inequality in premature mortality, these distributions alone do not seem to be able to fully explain mortality differences [2]. Thus, a lot of current research focuses on interactions between SES and risk factors and between different risk factors within socioeconomic strata.

Alcohol use is a major risk factor for premature mortality [3], globally and particularly in high- and middle-income countries, and the lower the age cutoff for the definition of premature mortality, the more important the impact of alcohol [4]. Specifically for alcohol use, evidence seems to indicate an overproportional impact in lower socioeconomic strata: despite higher rates of abstention in lower socioeconomic strata, alcohol-attributable mortality has been found to be higher, and this fact has been labelled the “alcohol harm paradox” by some researchers [5]. Riskier drinking patterns among drinkers of low SES, cumulative and interactive effects of other risk factors that cluster in people of low SES, and interactive effects between SES and alcohol use have been postulated as potential mechanisms underlying the increased mortality risk of people of low SES. However, empirical research on interactions between SES and alcohol use in relation to mortality or on 3-way interactions between SES, alcohol use, and other risk factors has been rare.

## SES, alcohol use, and cardiovascular mortality

In this regard, the paper of Degerud and colleagues in this week’s *PLOS Medicine* promises to become an important piece of the puzzle given its focus on cardiovascular mortality [6], globally the leading cause of death. Using a large population-based sample in Norway, they analyzed not only the associations between alcohol use and cardiovascular mortality by SES but also specifically the interaction between SES and alcohol use indicators. They found a significant interaction for drinking frequency and SES, but only a main effect for frequency of heavy drinking occasions with a nonsignificant interaction. Thus, the study observed that while heavy episodic drinking was associated with an elevated risk of cardiovascular mortality irrespective of SES, a moderate frequency of alcohol use was associated with more protective effects in the high compared with low or middle socioeconomic strata, and the protective effect of moderate drinking on cardiovascular mortality was not significant for people of low SES.

The lack of a significant interaction effect between heavy drinking occasions and SES on cardiovascular mortality comes as a surprise, as the drinking pattern of occasional heavy drinking has been suspected to be one of the main reasons for the differential effect of alcohol use in different socioeconomic strata. Three reasons may have contributed to this finding by Degerud and colleagues [6]: firstly, the sample size for many categories of higher frequency of heavy drinking was small; secondly, all alcohol use variables were measured only once in lifetime, and measurement of patterns of drinking is considerably less reliable than measurement of drinking status; and thirdly, the authors included systolic blood pressure, a variable on the causal pathway from alcohol use to cardiovascular events [7], as a potential confounding variable, thus reducing the effect size of the alcohol effect and decreasing the odds of finding an interaction effect.

## Implications for policy

Substance use in general and alcohol use in particular have been key to recent reversals of life expectancy [8] in high- and middle-income countries, and widening mortality inequalities play a key role here. For instance, Case and Deaton [9] showed that the decreasing life expectancy in middle-aged non-Hispanic whites in the United States was associated with increases in mortality inequalities in key causes of death such as poisoning, suicide, and liver cirrhosis. Life expectancy stagnates for the US as a whole, and again, substance use in lower socioeconomic strata seems to play a key role [10,11]. As in the paper of Degerud and colleagues [6], the same level of substance use was linked to higher mortality risks in individuals of lower SES compared to those of higher SES.

One main implication of this phenomenon states that it is not appropriate simply to extrapolate from risks associated with alcohol use in higher-income populations to address lower-income populations in which the impact of alcohol use is largest. As a consequence, alcohol policy measures not only need to be effective and cost-effective but also need to contribute to a reduction of health inequalities in alcohol-attributable mortality. Again, research on the impact of different alcohol policy measures on inequality needs to be specifically conducted; at this point, minimum unit pricing seems to be a measure that promises to reduce inequalities, albeit mainly based on mathematical modelling rather than on empirical evidence [12]. Theoretically, all measures that reduce the affordability of alcohol could be named here as promising. However, history of the recent decades has shown that affordability of alcohol has increased in almost all parts of the world (certainly in high- and middle-income countries) despite the implementation of more national alcohol policies [4]. In other words, different alcohol policy measures need to be implemented in the future guided by new empirical evidence.

## Abbreviations

SES: socioeconomic status
